# Women's Lives and Birth Experiences Are Important! Evaluation of Women's Perceptions of Respectful Maternal Care: The Mother‐Friendly Hospital Difference

**DOI:** 10.1111/jep.14316

**Published:** 2025-01-20

**Authors:** Elif Dağlı, Feyza Aktaş Reyhan, Betül Uncu

**Affiliations:** ^1^ Department of Health Care Services, Abdi Sütcü Vocational School of Health Services Çukurova University Adana Turkey; ^2^ Midwifery Department, Faculty of Health Sciences Kütahya University of Health Sciences Kütahya Turkey; ^3^ Midwifery Department, Faculty of Health Sciences İstanbul University‐Cerrahpaşa İstanbul Turkey

**Keywords:** midwife, mother, mother‐friendly hospital, respectful maternity care

## Abstract

**Aim:**

The aim of this study is to determine women's perceptions of respectful maternity care, the effect of giving birth in a mother‐friendly hospital on this perception and other factors affecting this perception.

**Background:**

The philosophy of a mother‐friendly hospital includes respectful maternity care. Few quantitative studies have been conducted in Turkey to assess the prevalence of respectful maternity care during childbirth and none have examined the difference between respectful maternity care in mother‐friendly and nonmother‐friendly hospitals.

**Methods:**

This descriptive and comparative study was conducted between December 2023 and September 2024 with 319 primiparous women who applied to the Obstetrics and Gynecology Outpatient Clinic of a mother‐friendly and a nonmother‐friendly hospital in Turkey for postpartum follow‐up. The data were collected face‐to‐face using the Descriptive Characteristics Form and Women's Perception of Respectful Maternity Care Scale. For data analysis, *χ* ^2^ and independent samples *t* test methods were used in SPSS 26 program.

**Results:**

In the study, a significant difference was obtained between the Perception of Respectful Maternity Care Scale scores of women according to the type of hospital where the birth was performed and the level of education (*p* < 0.05). The mean scale scores of women who gave birth in a mother‐friendly hospital and whose educational level was secondary education and above were higher than other women (*p* < 0.05). There was no significant difference between the scale scores of women according to the variables of age, employment status, place of residence, number of pregnancies and the last pregnancy being planned (*p* > 0.05).

**Conclusions and Implications for Practice:**

It was determined that women's perception of respectful maternity care was highly positive, and the factors affecting this perception were delivery in a mother‐friendly hospital and educational level. The current study may contribute to the development of policies for the dissemination of mother‐friendly practices and the provision of respectful birth services, and may also support efforts to improve the quality of care.

## 1 Introduction

The World Health Organization (WHO) emphasizes the importance of providing respectful, supportive and evidence‐based care to women during the birth process. These recommendations include respecting women's rights and protecting their privacy and autonomy. It is also stated that continuous emotional and physical support should be provided during labor and that women should be given the opportunity to have a supportive person by their side. Women's informed consent and active participation in decision‐making processes should be encouraged. For pain management, both pharmacological and nonpharmacological options should be offered in accordance with individual preferences, and the natural birth process should be supported by avoiding unnecessary medical interventions. These recommendations aim to create a supportive environment for women to have positive birth experiences and improve maternal and infant health [1]. Disrespectful, abusive or coercive approaches by providers in healthcare settings have been observed to have negative consequences for maternal and newborn health [2]. In this context, The International Federation of Gynecology and Obstetrics (FIGO) and other International partners have developed a strategy in response to these issues. This strategy includes the Mother and Baby Friendly Birth Facility Initiative [3]. This concept, which emerged in the 1990s, aims to improve the quality of maternity services and provide mothers with a more respectful and safe birth experience. The adoption of this concept by the WHO in 2003 led to the global dissemination of respectful birth care.

The aim of care in the mother‐friendly birth model is to support the improvement of maternal and child health through evidence‐based practices. By providing mothers with the necessary information and support during the birth process, this model promotes healthy birth experiences and strengthens the mother–infant bond [4]. Mother‐friendly hospitals (MFH) offer many important benefits that improve the health of mothers and babies. In these hospitals, mothers' breastfeeding rates increase, as well as normal delivery rates [5]. Moreover, in addition to providing improved health outcomes for both mothers and infants, these hospitals also offer psychological support to mothers, increasing their emotional well‐being [6]. In addition, the mother‐friendly care model and mother‐friendly programs are powerful responses to address problems in maternity care services, such as high cesarean section rates, low breastfeeding rates and women's rights issues. Today, the concept of “mother‐friendly hospital” is based on rights against abuse (physical, verbal, violation of privacy) and neglect in maternity services, especially in developing regions [7]. However, despite the success of this ideal at the global level, it has also been observed that practices vary at the local level. In particular, the implementation of MFH initiatives in some regions varies depending on the training of health professionals, infrastructure and local health policies. This is an important factor affecting the effectiveness of MFH practices, and suggests that they should be customized according to the needs of each hospital and community [8].

The global average cesarean section rate is 21.10% and 60.50% in Turkey [9], which is considerably higher than the WHO's ideal cesarean section rate of 10%–15% [10]. It is known that many factors play a role in this increase. However, when the reasons originating from health institutions are examined, it is noteworthy that factors such as inadequacies in preparing women for birth, inappropriate birth environment, unkind approaches of health professionals and inadequate support are the biggest factors [11, 12]. All this information shows that mothers are afraid of childbirth, do not trust healthcare professionals, prefer cesarean section birth away from normal birth, and have negative birth experiences when they prefer normal birth. At this point, it would be useful to discuss the benefits of MFH practice [12]). Another component of MFH practice is the provision of prenatal care and counseling services to expectant mothers. Expectant mothers should be able to contribute to birth planning. It is important that expectant mothers and their relatives are fully and accurately informed about the mode of delivery and its possible benefits and harms [13]. Respectful maternity care (RMC) plays an important role in improving the accessibility and quality of maternity services and focuses on the social and relational dimensions of maternity care [14]. Few quantitative studies have assessed the prevalence of RMC during childbirth in Turkey and none have examined the difference between RMC in mother‐friendly and non‐MFHs. Accordingly, the current study aims to determine women's perceptions of RMC, the effect of giving birth in an MFH on this perception, and other factors affecting this perception.

### 1.1 This Study Will Answer the Following Research Questions

- Is there a relationship between women's demographic characteristics (education level, age, employment status, etc.) and their perceptions of RMC?
- Does giving birth in an MFH have an effect on women's perceptions of RMC?

## 2 Materials and Methods

### 2.1 Research Type

This research is a descriptive comparative study. STROBE reporting guideline was taken into consideration in the study [15].

### 2.2 Research Sample

The study was conducted between December 2023 and September 2024 with women who came to the Obstetrics and Gynecology Outpatient Clinic of two hospitals in a mother‐friendly and nonmother‐friendly in a province in Turkey. The number of primiparous women who applied to the Obstetrics and Gynecology Outpatient Clinic in 2022 in the MFH was 2076, while the number of primiparous women in the non‐MFH was 1210. In the study, the sample size was calculated by using the sampling method with a known population. In the calculation of the sample size, the Raosoft program was used and the minimum sample size was calculated as 162 for the MFH and 135 for the non‐MFH with a Type I error of 0.05 and a test power of 0.80 (*α* = 0.05, 1 − *β* = 0.80). Considering the possible loss of cases, the study sample consisted of 319 (172 and 147) women determined according to the random sampling method.

#### 2.2.1 Inclusion Criteria

Primiparous women who received childbirth preparation training, had a vaginal delivery at term, presented to the delivery room in the latent phase, did not experience any pregnancy‐postpartum complications and volunteered to participate in the study were included in the study.

### 2.3 Data Collection Tool

The data of the study were collected with the Descriptive Characteristics Form and Women's Perception of RMC Scale.

#### 2.3.1 Descriptive Characteristics Form

The form prepared by the researchers consists of eight questions to examine the sociodemographic and obstetric characteristics of the participants [16, 17, 18, 19].

#### 2.3.2 Women's Perception of RMC Scale (WP‐RMC)

The scale was developed by Ayoubi et al. [20] to assess women's perception of RMC in Iran. Çamlıbel and Uludağ [21] adapted it into Turkish. The original scale consists of 19 items and three subscales and is scored on a 1–5 scale: 1 = almost never; 2 = sometimes; 3 = often; 4 = almost always; 5 = always. Items 15, 16, 17 and 19 are reverse scored. As a result of the internal consistency analysis of the scale, Cronbach's α coefficient was found to be 0.91. The minimum score is 19 and the maximum score is 95. Higher scores indicate a more positive perception of respectful maternal care.

### 2.4 Data Collection

The data of the study were collected face‐to‐face by the first researcher with women who came to the gynecology and obstetrics outpatient clinics of the two institutions where the study was conducted for postpartum control. After the participants were informed about the study, those who were willing to participate were interviewed. During data collection, care was taken to ensure that the participants were alone and away from distracting factors (pain, noise, crowd, baby's needs, etc.). It took approximately 10 min to fill out the form.

### 2.5 Ethical Procedure

For the implementation of the study, ethical approval was obtained from the Noninterventional Ethics Committee of a state university (decision dated 1 September 2023, numbered 136/70). Permission was obtained from the Provincial Health Directorate (decision dated 29 November 2023 and numbered E‐96172664‐050.06.04‐230513582) to collect data from hospitals. Verbal and written informed consent was obtained at the beginning of the survey form in line with the consent of the participants and from women who volunteered to participate in the study. The study was conducted in accordance with the ethical principles of medical research involving human subjects of the Declaration of Helsinki.

### 2.6 Data Analysis

SPSS 26 program was used for data analysis. Distribution according to demographic categorical variables was given and the proportions related to the types of hospitals where they gave birth and demographic variables were compared with the *χ* ^2^ analysis method. This method is a nonparametric method and compares the rates of two different categorical variables with at least two categories [22]. Outliers were controlled for the overall and subdimension scores of the women's perception of respectful maternal care scale. Tabachnick and Fidell [23] stated that if the z standard values of the measurements are outside the range of ±3.30, they are outliers. For the overall and subdimension scores of the respectful maternal care women's perception scale, the z value of four participants was < −3.30 and four participants were excluded. Skewness and kurtosis values were examined for the normality of the overall and subdimension scores of the perception of respectful maternal care women scale. George & Mallery [24] stated that if these values are between ±1, they are normally distributed. The overall and all subdimensions of the scale were found to be normally distributed, and the overall and subdimension scores of the scale of women's perception of RMC according to all demographic variables were compared using the independent samples *t* test method. The two basic assumptions of this method are the normality of the scores and the number of data in the groups should be large enough (*N* > 30) [22]. For statistical analyses, *p* < 0.05 significance level was examined.

## 3 Results

It was determined that 53.9% of the women gave birth in an MFH, 67.7% were 34 years of age or younger, 51.7% had secondary education and above (high school and above), 58.6% were employed and 58% lived in the city. It was also found that 61.1% of the women had income lower than expenditure and 65.2% had planned pregnancies in their last pregnancy (Table 1).

**Table 1:** Comparison of women's hospital preferences and some of their characteristics.

Variable	Group	Mother‐friendly hospital	Nonmother‐friendly hospital	Statistics	*p* value
		*N* (%)	*N* (%)		
Age	34 and below	112 (65.1)	104 (70.7)	1.15	0.284
	35 and above	60 (34.9)	43 (29.3)		
Education level	Primary education	80 (46.5)	74 (50.3)	0.465	0.495
	Secondary education and above	92 (53.5)	73 (49.7)		
Employment status	Working	105 (61.0)	82 (55.8)	0.905	0.341
	Not working	67 (39.0)	65 (44.2)		
Place of residence	Urban	108 (62.8)	77 (52.4)	3.526	0.06
	Rural	64 (37.2)	70 (47.6)		
Income level	Income lower than expenditure	101 (58.7)	94 (63.9)	0.911	0.34
	Income equal to expenditure and above	71 (41.3)	53 (36.1)		
Last pregnancy planned status	Planned	109 (63.4)	99 (67.3)	0.552	0.458
	Unplanned	63 (36.6)	48 (32.7)		

Among women who gave birth in an MFH, 65.1% were 34 years of age or younger and 34.9% were 35 years of age or older, while 70.7% of women who gave birth in other hospitals were 34 years of age or younger and 29.3% were 35 years of age or older. While 46.5% of women who gave birth in an MFH had primary education and 53.5% had secondary education, 50.3% of women who gave birth in other hospitals had primary education and 49.7% had secondary education. Sixty‐one percent of the women who gave birth in the MFH and 55.8% of the women who gave birth in the other hospital stated that they were employed. Among women who gave birth in an MFH, 62.8% stated that they lived in urban areas and 37.2% in rural areas, while 52.4% of women who gave birth in other MFHs stated that they lived in urban areas and 47.6% in rural areas. While 58.7% of women who gave birth in an MFH stated that their income was lower than their expenses and 41.3% stated that their income was equivalent to their expenses, 63.9% of women who gave birth in other hospitals stated that their income was lower than their expenses and 36.1% stated that their income was equivalent to their expenses. Among women who gave birth in an MFH, 63.4% stated that their most recent pregnancy was planned and 36.6% stated that it was unplanned, while 67.3% of women who gave birth in other hospitals stated that their most recent pregnancy was planned and 32.7% stated that it was unplanned. There was no significant relationship between all demographic characteristics of the women and the rates related to hospital preferences, and the groups were homogeneous and independent (*p *> 0.05) (Table 1).

The overall and subdimension scores of the WP‐RMC were obtained by summing the items. WP‐RMC overall scores were between 56 and 95 with a mean of 82.24. Comfort provision subscale scores were between 21 and 35 with a mean of 30.42; participatory care subscale scores were between 20 and 35 with a mean of 30.13 and maltreatment subscale scores were between 12 and 25 with a mean of 21.68. Skewness values were between −1.047 and −0.935 and kurtosis values were between 1.352 and 1.693 and all scale and subscale scores were normally distributed (Table 2).

**Table 2:** Descriptive statistics table of the general and subdimensions of the WPRMCS.

Scale	Min	Max	Mean	SD	Skewness	Kurtosis
Comfort provision	21	35	30.42	2.68	−1.009	1.693
Participative care	20	35	30.13	2.93	−0.964	1.489
Maltreatment	12	25	21.68	2.33	−0.935	1.352
WPRMCS total	56	95	82.24	7.69	−1.047	1.681

Abbreviation: WPRMCS, Women's Perception of Respectful Maternity Care Scale.

According to the type of hospital where the women gave birth, a significant difference was found between the total scores of WP‐RMC (*t* (317) = 4.497, *p* < 0.05) and the subdimension scores of providing comfort (*t* (317) = 4.232, *p *< 0.05), participatory care (*t* (317) = 4.68, *p* < 0.05) and perceptions of mistreatment (*t* (317) = 4.068, *p* < 0.05). The mean total score and all subdimension mean scores of the WP‐RMC of women who gave birth in an MFH were higher than those of women who gave birth in a non‐MFH (Table 3).

**Table 3:** Comparison of the total and subdimension scores of the WPRMCS according to the hospital where the women gave birth and some of its characteristics.

Group	*N*	Providing comfort	Participatory care	Mistreatment	WPRMCS total
		Mean ± SD	Mean ± SD	Mean ± SD	Mean ± SD
Hospital					
Mother‐friendly hospital	172	30.99 ± 2.08	30.82 ± 2.46	22.16 ± 1.84	83.98 ± 6.14
Nonmother‐friendly hospital	147	29.76 ± 3.11	29.33 ± 3.23	21.12 ± 2.7	80.2 ± 8.77
*t*		4.232	4.68	4.068	4.497
*p* value		0.000*	0.000*	0.000*	0.000*
Age					
34 and under	216	30.47 ± 2.74	30.19 ± 3.01	21.75 ± 2.41	82.41 ± 7.9
35 and over	103	30.32 ± 2.55	30.01 ± 2.77	21.54 ± 2.16	81.87 ± 7.26
*t*		0.473	0.512	0.738	0.584
*p* value		0.636	0.609	0.461	0.56
Level of education					
Primary education	154	29.98 ± 2.88	29.56 ± 3.22	21.42 ± 2.45	80.96 ± 8.29
Secondary education and above	165	30.84 ± 2.4	30.66 ± 2.53	21.93 ± 2.2	83.43 ± 6.91
*t*		−2.888	−3.389	−1.991	−2.898
*p* value		0.004*	0.001*	0.047*	0.004*
Working status					
Working	187	30.45 ± 2.59	30.28 ± 2.89	21.79 ± 2.24	82.53 ± 7.49
Not working	132	30.38 ± 2.8	29.92 ± 2.99	21.53 ± 2.46	81.83 ± 7.98
*t*		0.249	1.101	0.985	0.804
*p* value		0.804	0.272	0.325	0.422
Place of residence					
Urban	185	30.5 ± 2.55	30.33 ± 2.76	21.81 ± 2.26	82.64 ± 7.31
Rural	134	30.31 ± 2.84	29.86 ± 3.15	21.51 ± 2.42	81.69 ± 8.18
*t*		0.623	1.42	1.099	1.091
*p* value		0.534	0.157	0.273	0.276
Income level					
Income is lower than expenses	195	30.54 ± 2.72	30.24 ± 2.84	21.85 ± 2.36	82.63 ± 7.67
Income is equal to expenses and above	124	30.23 ± 2.61	29.96 ± 3.07	21.43 ± 2.27	81.62 ± 7.72
*t*		1.008	0.835	1.567	1.144
*p* value		0.314	0.404	0.118	0.254
Latest pregnancy planned status					
Planned	208	30.45 ± 2.69	30.23 ± 2.89	21.68 ± 2.35	82.36 ± 7.71
Unplanned	111	30.37 ± 2.66	29.95 ± 3.02	21.69 ± 2.3	82.01 ± 7.69
*t*		0.262	0.826	−0.058	0.388
*p* value		0.793	0.409	0.954	0.698

Abbreviations: t, Independent groups *t* test statistics; WPRMCS, Women's Perception of Respectful Maternity Care Scale.

* *p* < 0.05.

Significant differences were found between the total scores of WP‐RMC (*t* (317) = −2.898, *p* < 0.05) and the subdimension scores of providing comfort (*t* (317) = −2.888, *p* < 0.05), participatory care (*t* (317) = −3.889, *p* < 0.05) and perceptions of maltreatment (*t *(317) = −1.991, *p* < 0.05) according to the educational level of the women. The mean total score and all subdimension mean scores of WP‐RMC for women with secondary education and above were higher than those of women with primary education (Table 3).

There was no significant difference (*p* > 0.05) between the total scores and all subdimension scores of the WP‐RMC according to the variables of age, employment status, place of residence and planned last pregnancy (Table 3).

## 4 Discussion

In this study, women's perceptions of RMC, the effect of giving birth in an MFH on this perception and other factors affecting this perception were evaluated. It was determined that women's perceptions of RMC were highly positive, and the factors affecting this perception were giving birth in an MFH and educational level. In the present study, women who gave birth in an MFH and non‐MFH were similar in terms of sociodemographic and obstetric characteristics. Comparison between similar sample groups is important in revealing the perception of RMC [25].

The findings of the study show that women's perceptions of RMC are highly positive. These findings are consistent with similar studies in the literature. In particular, it was found that women who gave birth in MFHs perceived RMC more positively than those who gave birth in other hospitals [26, 27]. MFHs offer a more supportive environment by taking into account the needs of women during the birth process and this makes women's birth experiences more positive. In addition, it is clear from this study that educational level plays an important role in perceiving RMC. It was observed that women with higher levels of education viewed all aspects of care more positively, whereas women with lower levels of education had more negative experiences during the care process. These findings are supported by other studies [28, 29]. The fact that women with higher levels of education are more conscious and tend to defend their rights may lead to more positive perceptions of the care process, while women with lower levels of education may have more negative experiences.

In the study, perceptions of RMC differed significantly according to the types of health facilities women used for delivery. Women who gave birth in an MFH evaluated the perception of RMC more positively than those who gave birth in a non‐MFH. In MFHs where women experience RMC, being in supportive and harmonious environments that are suitable for the needs of women and their families significantly affects their hospital choices [30]. In a study, it was found that women who gave birth in MFHs had lower rates of restriction in movement and food intake and higher rates of meeting physical needs than those who gave birth in non‐MFHs [26]. In a quasi‐experimental study conducted in Taiwan, it was found that women received less epidural anesthesia and induction medication and obtained higher self‐reported satisfaction scores in hospitals where the MFH model was applied [27]. MFHs provide a supportive environment by prioritizing the needs of women during pregnancy and childbirth. These hospitals aim to provide a healthy experience for the woman, her baby and her family through services such as newborn care, breastfeeding education and family involvement. By encouraging emotional and information sharing between the woman and her family, they contribute to a healthier and more peaceful process. With this approach, MFHs strengthen the integrity of families by providing not only physical health services but also psychological support.

In the present study, women who gave birth in MFHs scored higher on the comfort provision subcomponent of RMC. In a study conducted in an MFH in Iran, it was reported that comfort provision methods such as companionship support and the use of upright positions during labor helped women to feel less labor pain [18]. In in‐depth interviews with women who experienced normal birth in non‐MFHs, women expressed that RMC was lacking, they could not give birth in the position they wanted, they did not receive companion support during the birth process, and many negative situations that were not suitable for RMC [31]. Women stated that they most wanted a companion in the hospital where they gave birth and that they should be allowed to move freely during the birth process [32]. These findings show that MFHs offer more comfort and support to women during the birth process, which positively affects their birth experiences, while in non‐MFHs, women face deficiencies and negativities in their care processes. Therefore, it is recommended that healthcare providers should make practices that respect women's rights, provide comfort and companion support more widespread during the birth process.

In this study, it was found that women who gave birth in non‐MFHs scored lower on the participatory care component. As found in the literature, mothers in non‐MFHs state that they want to have a say in their births [33]. Altman et al. [34] emphasized in their study that women want to be involved in the decision‐making process throughout their care experiences and that they highly value autonomy and decision‐making power during pregnancy and childbirth. Women reported feeling disempowered and frustrated when these rights were not granted to them as part of their relationship with healthcare providers, which decreased trust in healthcare providers and the healthcare system. The findings suggest that women's exclusion from decision‐making in non‐MFHs negatively affects their birth experiences and undermines their trust in health services; therefore, it is recommended that health institutions strengthen their practices to recognize women's autonomy and decision‐making rights.

In a study conducted in India, it was reported that women who received RMC were not mistreated, their birth experience improved and their overall satisfaction increased [35]. On the other hand, other studies in the literature reported that women were subjected to mistreatment during labor in non‐MFHs [17, 19]. In the current study, contrary to the literature, it was found that women who did not give birth in an MFH had low scores on the subcomponent of exposure to mistreatment. This finding is very important because it is the most fundamental right of women in all institutions to receive respectful care. While the mental health of women who give birth in MFHs is generally better protected, negative experiences in non‐MFHs can negatively affect women's intrapartum and postpartum mental health [36]. Women who have negative birth experiences are at increased risk of mental problems such as postpartum depression, which can make it difficult to participate in the workforce [33]. This situation highlights both women's right to a healthy birth experience and the need for equal opportunities to participate in the labor force. Therefore, promoting MFHs and preventing mistreatment are critical to improving women's birth experiences. It has been reported that birth takes place under optimum conditions and satisfaction is high in MFHs [37]. RMC aims to ensure that mothers' rights are respected and that they have a safe birth experience [38].

When women feel supported, respected and safe, and when they are enabled to participate in joint decision‐making processes with their care providers, they are more likely to have positive experiences [39]. Providing comfort, participatory care and mistreatment, which are components of RMC, are the main elements that shape women's experiences during the birth process. The impact of each of these components plays a critical role in determining the quality of maternity care. The philosophy of an MFH includes RMC [38]. Therefore, expanding MFH practices and shaping RMC according to the needs of women at every stage will improve birth experiences and increase women's trust in healthcare services.

### 4.1 Practical Recommendations for Future Research and Clinical Practice

The following recommendations can be taken into consideration for the effective provision of RMC:

#### 4.1.1 Education and Awareness

Continuous trainings for health professionals should provide empathetic care that respects women's rights. Continuous training programs should be organized for health professionals on RMC. These trainings can help develop skills in respecting women's rights, empathizing and providing nonviolent care.

#### 4.1.2 Policy Development

Establish hospital policies that promote RMC and standardize care processes. Policies that promote the implementation of RMC in hospitals and clinics need to be established. By standardizing care processes, these policies can help take concrete steps to prevent women from being mistreated during childbirth.

#### 4.1.3 Women's Participation and Information

Tools such as educational materials and birth plans should be provided to increase women's active participation in the birth process. Providing women with more information about the birth process encourages their active participation. Educational materials, counseling services and practices such as making a birth plan enable women to have more say in their own care.

#### 4.1.4 Supportive Environments

In MFHs, an environment where women can be comfortable and psychologically supported throughout the birth process should be created by strengthening psychological support services. Considering that birth is not only a physical but also an emotional and psychological process, it is important to strengthen psychological support services.

#### 4.1.5 Research

More research on the effects of respectful motherhood care is needed to identify the most effective strategies. To better understand the effects of RMC, more quantitative and qualitative research is needed. This research could help identify which strategies are most effective by analyzing differences between hospitals that implement respectful care and those that do not.

These recommendations will help to expand RMC and improve women's birth experiences.

## 5 Conclusions and Recommendations

This study revealed that women's perception of RMC was highly positive and the main factors affecting this perception were giving birth in an MFH and educational level. The implementation of MFH models only in secondary and tertiary healthcare services constitutes an important limitation for the dissemination of this model. However, considering the preventive and protective role of non‐MFH in pregnancy and postnatal follow‐up, it is of great importance to adopt the concept of mother‐friendly in primary healthcare. The evaluation of health services provided to women and their families within the framework of RMC should not be limited to the institution where the delivery takes place. Primary healthcare services have a critical importance in terms of protecting maternal health and improving the quality of postnatal care. Therefore, mother‐friendly practices need to be integrated into all health service delivery starting from primary care. In this context, it is emphasized that MFH practices should be expanded and RMC should be adopted by all healthcare providers in the development of health policies. In addition, the differences observed in women's perceptions of RMC according to their level of education reveal the importance of education and awareness‐raising programs to make health services more accessible and effective. In future research, it is recommended to conduct more in‐depth studies on how mother‐friendly care is implemented in different types of hospitals and primary healthcare services and the effects of these practices on women's birth experiences. In addition, developing policies for the dissemination of MFH practices and training all healthcare personnel on RMC will be important steps to improve women's health.

### 5.1 Limitations

This study has some limitations. First, only two different institutions (an MFH and a non‐MFH) were compared and only primiparous, vaginally delivered women were included. This may limit the validity of the findings of the study only on this group. Since the number of university and above graduates is quite low in the study, they were included in the secondary education and above education group. In addition, the sample consists only of women between the ages of 18–49. The selection of this age range may constitute a limitation in terms of generalizability of the results of the study, considering that the birth experiences of women in different age groups may differ. One of the biggest limitations of the study is that it is based on women's self‐reports. In particular, measuring women's perceptions of RMC based on self‐report may be limited to subjective perceptions.

### 5.2 Strengths of the Study

This study fills an important gap in the limited quantitative research on RMC in Turkey. The comparative analysis of MFHs contributes to the understanding of the impact of types of care on women's birth experiences and enables the development of recommendations for health policies. Moreover, the impact of demographic factors on perceptions of care makes the findings meaningful and provides concrete data to improve the quality of care. The fact that the data was not collected directly after delivery allowed mothers sufficient time to evaluate their birth environment and the care they received. To minimize participants' response bias, interviews were conducted in a private room and confidentiality was assured, which ensured the reliability of the data.

## Ethics Statement

Ethics approval was obtained from the Çukurova University Medical Faculty Ethics Committee (decision numbered 136/70, dated 1 September 2023).

## Conflicts of Interest

The authors declare no conflicts of interest.

## Data Availability

The data that support the findings of this study are available from the corresponding author upon reasonable request.

## References

[1] WHO , “WHO Recommendations: Intrapartum Care for a Positive Childbirth Experience,” 2018, https://www.who.int/publications/i/item/9789241550215. 30070803

[2] E. Yohannes , G. Moti , G. Gelan , D. K. Creedy , L. Gabriel , and C. Hastie , “Impact of Disrespectful Maternity Care on Childbirth Complications: A Multicentre Cross‐Sectional Study in Ethiopia,” BMC Pregnancy and Childbirth 24, no. 1 (2024): 380, 10.1186/s12884-024-06574-0. 38773395 PMC11110437

[3] S. Miller and A. Lalonde , “The Global Epidemic of Abuse and Disrespect During Childbirth: History, Evidence, Interventions, and FIGO's Mother‐Baby Friendly Birthing Facilities Initiative,” International Journal of Gynaecology and Obstetrics 131, no. 1 (2015): 49–52, 10.1016/j.ijgo.2015.02.005. 26433506

[4] N. Bolsoy , E. Bozhan‐Tayhan , S. Köken‐Durgun , E. Damar , and E. Kayıp , “The Knowledge and Attitudes of Health Professionals Working in Mother‐Friendly Hospitals About Complementary Therapy and Supportive Care Methods,” European Journal of Midwifery 6 (2022): 1–8, 10.18332/ejm/146166. PMC9006184 35509982

[5] J. Mondkar , D. Chawla , R. C. Sachdeva , et al., “Impact of Mother‐Baby Friendly Initiative Plus Approach on Improving Human Milk Feeding for Neonates in Hospital: A Quality Improvement Before‐and‐After Uncontrolled Study,” European Journal of Pediatrics 181, no. 1 (2022): 107–116, 10.1007/s00431-021-04141-9. 34216269

[6] H. H. Shao , S. C. Lee , J. P. Huang , and L. C. Hwang , “Prevalence of Postpartum Depression and Associated Predictors Among Taiwanese Women in a Mother‐Child Friendly Hospital,” Asia Pacific Journal of Public Health 33, no. 4 (2021): 411–417, 10.1177/10105395211001172. 33715458

[7] N. Erbaydar , “Mother‐Friendly Hospital Programme of Turkey: National Intervention to Improve the Quality of Maternity Services,” Eastern Mediterranean Health Journal 27, no. 2 (2021): 202–210. 33665805 10.26719/emhj.20.138

[8] N. Bolsoy , M. Gulsen , and B. C. Yavas , “A Cross‐Sectional Study of Mother‐Friendly Hospital Initiatives in Turkey: The Obstetricians and Midwives’ Views,” Journal of Midwifery & Reproductive Health 8, no. 4 (2020): 2463–2471.

[9] WHO , “Caesarean Section Rates Continue to Rise, Amid Growing Inequalities in Access,” 2021, https://www.who.int/news/item/16-06-2021-caesarean-section-rates-continue-to-rise-amid-growing-inequalities-in-acces.

[10] WHO , “Statement on Caesarean Section Rates,” 2015, https://apps.who.int/iris/bitstream/handle/10665/1.

[11] Ş. Birinci and Ü. M. Parpucu , “When a Caesarean Section Is Necessary: Analysis of Cesarean Sections Performed in the Republic of Turkey in 2022 in Accordance With the World Health Organization Multi‐Country Research Guidelines,” Journal of Turkish Society of Obstetric and Gynecology 20, no. 3 (2023): 184–190. 10.4274/tjod.galenos.2023.35919 PMC10478727 37667478

[12] P. Serçekuş‐Ak , O. Vardar , and S. Özkan , “Anne dostu hastanelerin yayginlaşmasi türkiye için neden önemlidir?için neden önemlidir?,” Necmettin erbakan üniversitesi sağlik bilimleri fakültesi dergisi 1, no. 1 (2018): 25–29.

[13] Ministry of Health , Public Hospitals General Directorate Health Services Department, 2016, https://khgmsaglikhizmetleridb.sa.glik.gov.tr/TR-42835/anne-dostu-hastane-listesi.html.

[14] S. Haghdoost , M. Iravani , A. H. Rahmani , and S. Montazeri , “Midwives' Experience of Respectful Maternity Care (RMC) Globally: A Meta‐Synthesis,” Nursing Ethics 31, no. 5 (2024): 951–979. 38113636 10.1177/09697330231218346

[15] S. Cuschieri , “The STROBE Guidelines,” Saudi Journal of Anaesthesia 13, no. 1 (2019): 31–34. 10.4103/sja.SJA_543_18 PMC6398292 30930717

[16] F. Aktas Reyhan , F. D. Sayiner , and H. Ozen , “A Mixed‐Design Study on the Development of Birth Unit Assessment Scale,” Midwifery 123 (2023): 103708. 37207495 10.1016/j.midw.2023.103708

[17] B. Birie and W. Niguse , “Experience of Respectful Maternity Care During Childbirth and Associated Factors in Public Hospitals of the South West Region of Ethiopia: An Institution‐Based, Cross‐Sectional Study,” BMJ Open 13, no. 7 (2023): e066849, 10.1136/bmjopen-2022-066849. PMC10347517 37433724

[18] S. Makvandi , K. Mirzaiinajmabadi , N. Tehranian , H. Esmily , and M. Mirteimoori , “The Effect of Normal Physiologic Childbirth on Labor Pain Relief: An Interventional Study in Mother‐Friendly Hospitals,” Maedica 13, no. 4 (2018): 286–293, 10.26574/maedica.2018.13.4.286. 30774727 PMC6362879

[19] B. Rajkumari , N. Devi , J. Ningombam , and D. Ingudam , “Assessment of Respectful Maternity Care During Childbirth: Experiences Among Mothers in Manipur,” Indian Journal of Public Health 65, no. 1 (2021): 11–15, 10.4103/ijph.IJPH43019. 33753683

[20] S. Ayoubi , F. Pazandeh , M. Simbar , M. Moridi , E. Zare , and B. Potrata , “A Questionnaire to Assess Women's Perception of Respectful Maternity Care (WP‐RMC): Development and Psychometric Properties,” Midwifery 80 (2020): 102573. 31734587 10.1016/j.midw.2019.102573

[21] M. Çamlibel , E. Uludağ , and F. Pazandeh , “Psychometric Evaluation of the Women's Perception of Respectful Maternity Care Scale Turkish Version,” Women & Health 62, no. 8 (2022): 700–710. 36071565 10.1080/03630242.2022.2118407

[22] J. Pallant , SPSS Survival Manual: A Step by Step Guide to Data Analysis Using SPSS for Windows (Version 12), 2nd ed. (New York: Open University Press, 2007).

[23] B. G. Tabachnick and L. S. Fidell , Using Multivariate Statistics (Boston: Pearson, 2013).

[24] D. George and M. Mallery , SPSS for Windows Step by Step: A Simple Guide and Reference, 10a ed. (Boston: Pearson, 2010).

[25] L. N. Namusonge and J. O. Ngachra , “Respectful Maternity Care Interventions: A Systematic Literature Review,” East African Journal of Health and Science 3, no. 1 (2021): 45–58.

[26] Z. Bilgin , “Anne dostu hastane modeli ve annelerin doğum memnuniyetleri,” Dokuz eylül universitesi hemşirelik fakültesi elektronik dergisi 15, no. 3 (2022): 279–288, 10.46483/deuhfed.892932.

[27] Y. P. Li , C. H. Yeh , S. Y. Lin , et al., “A Proposed Mother‐Friendly Childbirth Model for Taiwanese Women, the Implementation and Satisfaction Survey,” Taiwanese Journal of Obstetrics & Gynecology 54, no. 6 (2015): 731–736, 10.1016/j.tjog.2015.10.009. 26700994

[28] K. Afsana and S. F. Rashid , “The Challenges of Meeting Rural Bangladeshi Women's Needs in Delivery Care,” Reproductive Health Matters 9, no. 18 (2001): 79–89. 11765404 10.1016/s0968-8080(01)90094-1

[29] C. A. Moyer , P. B. Adongo , R. A. Aborigo , A. Hodgson , and C. M. Engmann , “‘They Treat You Like You Are Not a Human Being’: Maltreatment During Labour and Delivery in Rural Northern Ghana,” Midwifery 30 (2014): 262–268. 23790959 10.1016/j.midw.2013.05.006

[30] S. Özmen and S. Koyuncu , “Dost hastane kavrami ve türleri üzerine sistematik bir derleme,” Hacettepe sağlik idaresi dergisi 26, no. 3 (2023): 723–744.

[31] F. Aktaş‐Reyhan and E. Dağlı , “Kadınların saygili annelik bakimi doğrultusunda doğum deneyimlerinin değerlendirilmesi: Nitel bir araştirma,” Sakarya üniversitesi holistik sağlik dergisi 6, no. 1 (2023): 124–141, 10.54803/sauhsd.1228231.

[32] A. Yeşiltaş , Ş. D. Kaya , A. Yüceler , H. Görkemli , and G. Eren , “Hasta Gözüyle Anne Dostu Hastane Kriterleri,” Journal of Healthcare Management and Leadership 1 (2023): 1–13, 10.35345/johmal.1166937.

[33] M. A. Bohren , G. J. Hofmeyr , C. Sakala , R. K. Fukuzawa , and A. Cuthbert , “Continuous Support for Women During Childbirth,” Cochrane Database of Systematic Reviews 7, no. 7 (2017): CD003766, 10.1002/14651858.CD003766.pub6. 28681500 PMC6483123

[34] M. R. Altman , M. R. McLemore , T. Oseguera , A. Lyndon , and L. S. Franck , “Listening to Women: Recommendations From Women of Color to Improve Experiences in Pregnancy and Birth Care,” Journal of Midwifery & Women's Health 65, no. 4 (2020): 466–473. 10.1111/jmwh.13102 32558179

[35] H. Ansari and R. Yeravdekar , “Respectful Maternity Care During Childbirth in India: A Systematic Review and Meta‐Analysis,” Journal of Postgraduate Medicine 66, no. 3 (2020): 133–140, 10.4103/jpgm.JPGM_648_19. 32675449 PMC7542060

[36] S. Coo , M. I. García , and A. Mira , “Examining the Association Between Subjective Childbirth Experience and Maternal Mental Health at Six Months Postpartum,” Journal of Reproductive and Infant Psychology 41, no. 3 (2023): 275–288. 34672883 10.1080/02646838.2021.1990233

[37] P. S. Ak , O. Vardar , and S. Özkan , “Anne dostu hastanelerin yayginlaşmasi türkiye için neden önemlidir? *Necmettin erbakan üniversitesi sağ* ,” lık bilimleri fakültesi dergisi 1, no. 1 (2018): 25–29.

[38] M. Oveysi and S. E. Apay , “Doğumda algilanan destekleyici bakim ve memnuniyet düzeyi arasindaki ilişkinin belirlenmesi,” Genel tip dergisi 31, no. 3 (2021): 232–238.

[39] E. Özcan and A. Akdemir , “Bakımda gelişen bir paradigma: saygili annelik bakimiannelik bakimi,” Kadın sağlığı hemşireliği dergisi 9, no. 1 (2023): 56–61.

